# Plant Parasites under Pressure: Effects of Abiotic Stress on the Interactions between Parasitic Plants and Their Hosts

**DOI:** 10.3390/ijms22147418

**Published:** 2021-07-10

**Authors:** Lyuben Zagorchev, Wolfgang Stöggl, Denitsa Teofanova, Junmin Li, Ilse Kranner

**Affiliations:** 1 Zhejiang Provincial Key Laboratory of Plant Evolutionary Ecology and Conservation, Taizhou University, Taizhou 318000, China; lijmtzc@126.com; 2 Faculty of Biology, Sofia University “St. Kliment Ohridski”, 8 Dragan Tsankov Blvd., 1164 Sofia, Bulgaria; teofanova@biofac.uni-sofia.bg; 3 Department of Botany and Center for Molecular Biosciences Innsbruck (CMBI), University of Innsbruck, Sternwartestraße 15, 6020 Innsbruck, Austria; wolfgang.stoeggl@tirol-kliniken.at (W.S.); ilse.kranner@uibk.ac.at (I.K.)

**Keywords:** abiotic stress, biotic stress, drought, herbicide, parasitic plants, salinity

## Abstract

Parasitic angiosperms, comprising a diverse group of flowering plants, are partially or fully dependent on their hosts to acquire water, mineral nutrients and organic compounds. Some have detrimental effects on agriculturally important crop plants. They are also intriguing model systems to study adaptive mechanisms required for the transition from an autotrophic to a heterotrophic metabolism. No less than any other plant, parasitic plants are affected by abiotic stress factors such as drought and changes in temperature, saline soils or contamination with metals or herbicides. These effects may be attributed to the direct influence of the stress, but also to diminished host availability and suitability. Although several studies on abiotic stress response of parasitic plants are available, still little is known about how abiotic factors affect host preferences, defense mechanisms of both hosts and parasites and the effects of combinations of abiotic and biotic stress experienced by the host plants. The latter effects are of specific interest as parasitic plants pose additional pressure on contemporary agriculture in times of climate change. This review summarizes the existing literature on abiotic stress response of parasitic plants, highlighting knowledge gaps and discussing perspectives for future research and potential agricultural applications.

## 1. Introduction to Parasitic Flowering Plants

Parasitic flowering plants comprise a group of an estimated 4000 species in more than 20 plant families, or approximately 1.5% of the known vascular plant species [1]. These highly specialized plants are characterized by partial or complete loss of photosynthetic ability and depend on their hosts for photosynthates, mineral nutrients and water [2]. Parasitic plants are classified into two major categories. Hemiparasites contain chlorophyll and are able to photosynthesize. They obtain water and mineral nutrients from the host. Holoparasites are non-photosynthetic. They are obligate parasites, depending completely on the host [3,4]. Of all parasitic plants, relatively few species—less than 400—are holoparasites [5]. Parasitic plants attach to the host plants and absorb nutrients through haustoria, well-defined structural and physiological links with the host. Haustoria may vary in structure among parasitic plant families but remain a common feature of all of them (see [6] for a comprehensive overview of different haustoria types). Despite several anatomical and developmental differences, haustoria establish direct connection between the xylem and/or the phloem of the host and the parasite, to provide bidirectional flow of water, minerals and macromolecules including proteins, mRNAs [7,8] and genetic material, enabling horizontal gene transfer [9,10]. To facilitate this flow, parasitic plants tend to maintain lower water potential and higher transpiration rates in comparison to their hosts. As discussed below, the haustorial connection may also contribute to the exchange of stress-responsive molecules and potentially harmful compounds such as heavy metals. Depending on the attachment site, parasitic plants are categorized into root and stem parasites [6]. Throughout the literature, the term shoot parasites is synonymously used for stem parasitic plants [11]. Stem parasites are plants that attach and form haustoria into aerial host organs—either stem, leaf petiole or leaf surface.

Plant-plant parasitism evolved independently at least 12 or 13 times [1], facilitated by the formation of haustoria. Simultaneously, the transition from hemiparasitism to holoparasitism resulted in significant gene loss, especially in the plastid genome [2]. The mitochondrial genome was also subject to gene loss in at least some parasitic plants [12]. Compared to around 160 kbp of the fully functional tobacco plastome, some parasitic plants may have as little as 11–15 kbp [13] or even none [14].

Some parasitic plants are significant agricultural pests. Several species of dodders (*Cuscuta* spp.), witchweed (*Striga* spp.) and broomrapes (*Orobanche* spp.) cause serious damage to crop plants worldwide [15]. Dodders parasitize and cause yield losses mainly of alfalfa (*Medicago sativa* L.) and sugar beet (*Beta vulgaris* L.) but also other eudicot crop plants such as carrot (*Daucus carota* (Hoffm.) Schübl. & G. Martens), pepper (*Capsicum annuum* L.) or potato (*Solanum tuberosum* L.), etc. *Orobanche* spp. infect a variety of eudicot crops including carrot, sunflower, legumes and several species in the Solanaceae. Several Orobanche species cause significant agricultural losses and can be responsible for over 50% yield reduction, especially in combination with drought [15]. Another member of the Orobanchaceae, *Striga* spp., and especially *Striga hermonthica* (Delile) Benth., are major parasites of cereal crops and could account for annual losses of over $1 billion globally [16]. Research efforts are largely focused on identifying host resistance mechanisms [17], selection of parasite-resistant varieties of existing crop plants [18,19] and development of efficient control strategies [20]. However, parasitic plants are also important members of natural plant communities, which they can shape to a significant degree [21]. By selectively foraging on heterogeneous plant communities, parasitic plants suppress the growth of certain plant species, which benefits others and impacts on biodiversity [22,23].

The parasitic plant-host interactions, both in natural habitats and agricultural lands, is likely affected by abiotic stress. Abiotic stress factors, i.e., stress factors resulting from non-living factors [24], generally affect plant life, shape ecosystems and decrease agricultural production globally. Major abiotic stress factors are water stress, including drought and flooding, fluctuations or extremes of temperature or irradiation, salinity, mineral deficiency and toxic substances such as heavy metals, air pollutants or pesticides. Worldwide, approximately 96.5% of land area is affected by individual or multiple abiotic stress factors to a different extent [25], restricting or modifying plant growth and development. Parasitic plants are no less affected by abiotic stress factors, either directly or through the host. The aim of the present review is to summarize the scarce existing reports on the effects of abiotic stress on parasitic plants and the interaction with their hosts, highlighting the need for further research to (1) increase basic knowledge of host-parasite interactions under abiotic stress and (2) to better understand the potentially combined effects of abiotic stress in conjunction with parasite pressure on agriculture.

## 2. Possible Effects of Abiotic Stress on Parasitic Plants

**Figure 1:**
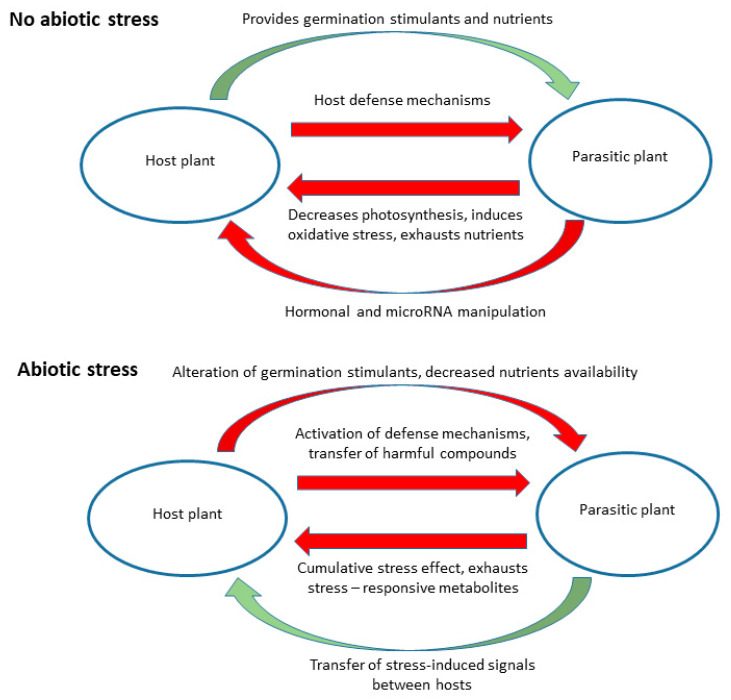
Possible effects of abiotic stress factors on the interactions between parasitic plants and their hosts. Green arrows represent beneficial interaction and red arrows represent harmful effects.

Parasitic plants may be affected by abiotic stress factors in a similar way as their hosts, e.g., by constraints of seed germination and seedling development due to drought and/or salinity, or indirectly, i.e., due to host-related constraints. The latter is especially true for holoparasites, most of which have no or only limited soil contact and lack photosynthesis. The possible effects of abiotic stress on host-parasitic plant interactions are summarized in Figure 1.

### 2.1. Effects through Alteration of Germination Stimulants, Released by the Host

An important adaptive strategy of root holoparasites is seed dormancy, preventing germination and seedling emergence in the absence of a suitable host. Germination occurs only in the presence of specific chemical compounds released by a potential host plant [26]. The best-studied germination stimulants are the strigolactones, confirmed to be essential for germination of members of the Orobanchaceae family, *Striga* spp. and *Orobanche* spp. [27]. For comprehensive review of the roles of strigolactones on host-parasitic plant interactions see Yoneyama et al. [28] and Teofanova et al. [29]. In brief, strigolactones, which are derived from carotenoids, are released by host roots to attract arbuscular mycorrhizal fungi but are also perceived by seeds and seedlings of parasites. In some cases, the level of recognition is so specialized that only a particular strigolactone is able to induce germination. For example, whereas several strigolactones, released by a wide variety of hosts, stimulate germination of multiple *Orobanche* spp., *O. cumana* is insensitive to them and requires dehydrocostus lactone, released by sunflower roots to germinate [30].

Strigolactone synthesis and release is needed for and regulated by arbuscular mycorrhiza symbiosis but also by different abiotic stress factors quantitatively and qualitatively. Numerous reports indicated negative effects of both salt and drought stress on strigolactone biosynthesis and release into root exudates, although strigolactone synthesis increased under stress in the presence of the fungal symbiotic partner [31,32]. Mycorrhizal symbiosis decreased strigolactone production under optimal conditions in tomato roots [33], impacting negatively on *Orobanche ramosa* seed germination. Negative effects of mycorrhizal symbiosis were also found in various *Orobanche* spp. parasitizing pea [34]. By contrast, the combination of salt stress and mycorrhizal symbiosis of the potential host improved germination of parasite seeds. This was clearly shown for the interaction between *Lactuca sativa* L. (host)–*O. ramosa* (parasitic plant)–*Glomus intraradices* Schenk and Smith (mycorrhizal fungus) [31]. Therefore, the release of signaling molecules, and particularly strigolactones, appears to depend on both abiotic and biotic factors and strongly affects at least some parasitic plants.

### 2.2. Host Biomass and Health Status

Parasitic plants are highly dependent on host availability, and abiotic stress factors potentially reduce this availability. Therefore, host selection is of crucial importance to the success of parasitic plants under non-optimal conditions. The impressive seed dormancy and longevity of root holoparasites [35], in which the lack of seed storage compounds and its own photosynthates does not allow seedling growth in the absence of a suitable host, might be of crucial importance to cope with the issue of host selection. Apart from germination stimulants, some parasitic plants, such as dodders, appear to have highly specialized mechanisms of host localization and selectivity. It was suggested that members of the *Cuscuta* genus use both chemical signals, such as the terpenoids α-pinene and β-myrcene [36] and light signals [37] to sense their potential hosts. *Cuscuta campestris* seedlings were shown to selectively infest hosts with high chlorophyll contents, which they detect via the low-red to far-red ratio of transmitted light [37]. In the salt-sensitive model plant *Arabidopsis thaliana* (L.) Heynh, chlorophyll contents decreased when the plant was grown under saline conditions as compared to its salt-tolerant relative *Eutrema halophilum* (C.A. Mey.) Al-Shehbaz & S.I. Warwick [38]. Consequently, it could be assumed that a parasite would preferentially attach to a stress-tolerant host, which would produce more photosynthates, and thus more biomass. Some reports confirm this assumption, e.g., dodders appear to be able to sense host quality, possibly via multiple mechanisms, and avoid unsuitable ones even before the formation of haustoria [39].

However, host “quality” may not simply depend on biomass availability. When exposed to abiotic stress factors such as drought and salinity, hosts can accumulate higher concentrations of compatible solutes, potentially resource-rich substrates for parasites, which may even enhance host quality compared to non-stressed hosts [40]. According to the case study of the *Arabidopsis–Eutrema* pair under salt stress, the salt-tolerant *Eutrema* showed much better relative growth rates (e.g., more biomass available to a potential parasite) under saline conditions [41]. However, treatment with up to 200 mM NaCl led to much higher concentrations of leaf proline in *Arabidopsis*. Higher NaCl concentrations proved detrimental to *Arabidopsis*, whereas *Eutrema* still grew on substrate containing up to 500 mM NaCl, with a gradual increase in proline contents. Therefore, it appears that a stress-sensitive host may be of better “quality” at lower stress levels and becomes a “worse” host or does not grow (e.g., is not available) when stress levels increase with higher salt concentrations.

### 2.3. Effects of the Host Defense System

As do pathogens and herbivores, parasitic plants also induce defense mechanisms in the parasitized host. The best-studied responses involve jasmonic acid (JA) and salicylic acid (SA)-induced systemic acquired resistance (SAR) responses, also involving the expression of pathogenesis related (PR) genes [42]. In addition, abscisic acid (ABA)-mediated responses [17] were also established. The same hormones are also important players response to abiotic stress factors, thus accounting for several response mechanisms common to both abiotic and biotic stresses [43]. Evidence is accumulating that plants, weakened by abiotic stress factors become more vulnerable to biotic stress factors [44,45], yet to be confirmed for the interaction between hosts and parasitic plants. Conversely, under conditions of abiotic stress, parasitic plants could face potential hosts with already activated SAR. Several reports support this view that abiotic stress-induced responses could render potential host plants less susceptible or even insensitive to parasitic plant infection. Treatment of *Beta vulgaris* with NaCl had a dose-dependent effect on *Cuscuta salina* fecundity [40], suggesting that under moderate salinity the defense mechanisms of the host were harmful to the parasite, whereas at more saline conditions the parasite was able to be more successful despite its lower germination rate and lower host biomass availability. However, the abiotic stress resistance of certain cultivars may also be related to resistance to parasitic plants. This was shown in salt-tolerant *Vicia faba* L. cultivars, whose tolerance correlated with their resistance to *Orobanche* [46]. Resistance to parasitic plants is often related to decreased cell wall permeability, induced by protein cross-linking and callose and suberin deposition [47], factors that are also induced by abiotic stress factors. Therefore, at least in some cases, greater abiotic stress tolerance may result in enhanced resistance to parasitic plants due to the upregulation of defense mechanisms shared between abiotic and biotic stress responses. This assumption is supported by the abiotic stress-induced pathogen resistance reported for barley [48]. Cross-tolerance was mediated by a variety of SA, JA, ethylene, ABA and auxin-mediated signaling pathways and/or redox signaling, which, induced by a single stress factor, lead to enhanced resistance to multiple biotic and abiotic factors [49]. Therefore, for an assessment of the effects of the host’s defense mechanisms, host and parasite species and the specific circumstances need to be carefully considered.

### 2.4. Transmission of Harmful Compounds from Host to Parasite

The haustorial connection between parasitic plants and their hosts is a site of extensive, bidirectional exchange of water, mineral nutrients, organic compounds and macromolecules [8], although a certain degree of selectivity exists [6,50]. The capability to tap into the nutrient supply of the host strongly depends on the lower water potential of the parasite, which in turn depends on the concentrations of particular compounds in the host tissue [6]. Together with the translocation of nutrients from host to parasite, potentially harmful substances, or even pathogens, may also move through haustoria and will cause stress in the parasite. For example, herbicides were found to be transmitted by the host to *Cuscuta campestris* [51]. Other reports on potentially harmful compounds transmitted from the host to the parasite include those on heavy metals [52,53] as well as Na and Cl ions in the case of salt stress [54]. Recently, selectivity for transport of mineral elements through the haustoria was demonstrated in *Cuscuta reflexa* Roxb. [50], which suggests that some parasites might be able to exclude harmful elements. The accumulation of harmful elements in the parasite seems to be dependent on host species and environmental conditions (Table 1). In summary, a parasitic plant can experience abiotic stress factors indirectly, through the host.

## 3. Response to and Tolerance of Abiotic Stress Factors in Parasitic Plants

Assuming that the distribution of parasitic plants is defined by the availability of suitable hosts [64], it could be expected that parasitic plants are present mainly under favorable conditions. Indeed, the diversity of parasitic plant species is far greater in tropical and temperate climates and decreases significantly towards the sub-polar regions and in arid lands (for comprehensive overview see [65]). However, many parasitic plants are found in saline coastal lands, deserts and alpine or arctic climates, which are largely defined by the presence of stress tolerant hosts, adapted to grow and reproduce under suboptimal conditions. Table 1 provides an overview of parasitic plants occurring in extreme environments.

### 3.1. Drought Stress

Xerophytic root and stem parasites occur in numerous arid areas. The Sonoran Desert in Mexico and South-Western USA is especially rich in unique parasites [65]. *Cistanche phelypaea* (L.) Cout. is found in sand dunes in the Arabian Peninsula and *Amyema fitzgeraldii* (Blakely) Danser is known from arid areas of Western Australia. There are many examples of parasites occurring on xerophytic hosts including cacti and euphorbias, e.g., *Plicosepalus acaciae* (Zucc.) Wiens & Polhill parasitizing on *Euphorbia cactus* Ehrenb. ex Boiss, but only a few parasites were tested for xerophytic features themselves [65]. The mistletoe *Tristerix aphyllus* (DC.) Barlow & Wiens [66] is a cacti specialist. These examples are of rare, specifically adapted parasites without substantial agricultural impact, but evidence of economically important weeds such as *Orobanche cernua*, parasitizing on xerophytes, also exists [57].

**Figure 2:**
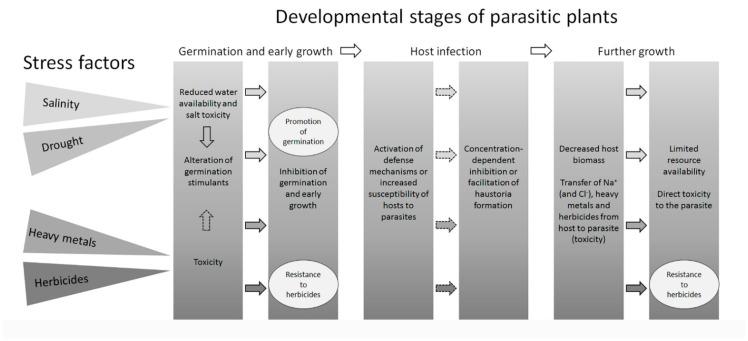
Summary of the effects of abiotic stress factors on different developmental stages of parasitic plants. Main stress factors and their effects are shown in different shading (not reflecting their severity). Dotted arrows depict suggested effects (to be confirmed experimentally). Ellipses denote exceptions to the rule. Furthermore, no reports were found that confirm that heavy metals or herbicides alter the production of strigolactones, but such an effect is not unlikely. No reports on the effects of abiotic stress factors on haustoria formation were found. Topical literature is cited in the text.

When considering the effects of drought stress on parasitic plants, it is important to note that especially holoparasites acquire water mainly from the host. Therefore, with the exception of seed germination and early seedling growth towards the host, the effects of drought on parasitic plants are mainly indirect, through the host. As expected, decreasing water potential affects both germination and early seedling growth negatively (Figure 2), shown for the root parasites *Orobanche crenata* [67], *Striga hermonthica* and *Alectra vogelii* Benth. [68]. However, Gibot-Leclerc [69] reported low sensitivity of *Orobanche ramosa* seeds to low water potential, similar to *Orobanche aegyptiaca* [70]. These apparent contradictions may be due to the different experimental conditions used in the above studies and care should be taken in the interpretation of the results. However, if these contradictory results could be confirmed, one might speculate that root parasites can germinate under limited water availability in the presence of a suitable host, allowing them to grow readily after infection. This possibility is supported by work reporting that the release of strigolactones from the host, needed for parasite seed germination, also altered by drought stress and can increase in mycorrhized and decrease in non-mycorrhized plants [32]. Furthermore, a recent report identified the *OaMAX2* gene in *Orobanche aegyptiaca* as a potential candidate to confer drought tolerance to the parasite [71]. Considering that the product of *OaMAX2* is part of the strigolactone signaling pathway, it seems that the host availability simultaneously triggers germination and drought response in this parasite, common in arid lands.

Another parasitic plant, the stem parasite *Cuscuta australis* R. Br., was reported to be insensitive to abscisic acid-mediated drought stress responses due to lost or non-functional ABA receptors [72]. In response to exogenous ABA, the parasite did not show inhibition of germination or suppression of hypocotyl elongation, defense mechanisms, observed in non-parasitic plants. This might be detrimental under drought but also ensures that even under suboptimal conditions the parasite will be able to grow towards potential hosts. On the other hand, Qin et al. [73] reported a several-fold increase in ABA content of dehydrated stems of *Cuscuta reflexa*, very similar to an ordinary response of non-parasitic plants. It remains to be clarified whether ABA-related response is species-specific within the *Cuscuta* genus. Nonetheless, stem parasitic plants may be indirectly affected by drought stress incurred by their hosts, which can decrease a host’s growth rate, thereby limiting the resources available to the parasite, as reported for the stem parasite *Cuscuta gronovii* Willd. ex Schult.-*Verbesina alternifolia* (L.) Britton ex Kearney [74] and *Amyema miquelii* (Lehm. ex Miq.) Tiegh.-*Eucalyptus largiflorens* F.Muell. [75] parasite-host pairs. The availability of drought-adapted hosts, however, means that parasitic plants could successfully thrive under drought conditions and may not need to evolve their own xerophytic features.

### 3.2. Salt Stress

**Figure 3:**
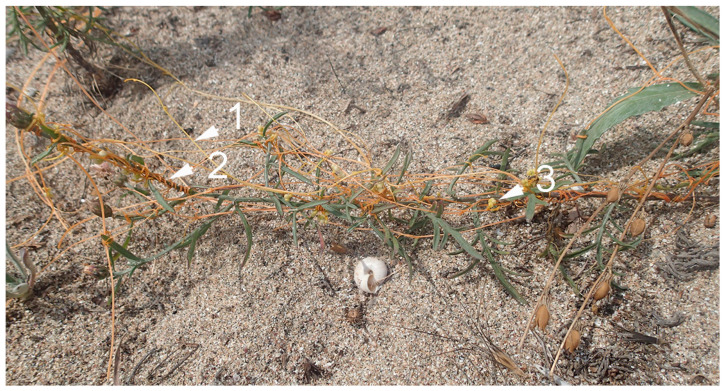
*Cuscuta campestris*, parasitizing *Centaurea arenaria* on a sand dune. Bulgarian Black Sea coast, 42°13′46″ N; 27°46′32″ E, July, 2018. White arrows show (1) the parasite stems, (2) site of infection and (3) inflorescence.

Parasitic plants also occur in saline coastal areas. At least four *Cuscuta* species are known to thrive under saline conditions: *C. sandwichiana* Choisy in Hawaii, *C. tasmanica* Engelm. in Tasmania and Southern Australia [76], *C. europaea* L. spp. halophyta (Fr.) Hartm. in Russia and Southern Scandinavia and *C. salina* Engelm. in North America. The latter mainly parasitizes on halophytes such as *Salicornia virginica* L. and *Frankenia salina* (Molina) I. M. Johnst., although it is not restricted to them [77]. A similar report highlighted the importance of *Cuscuta salina* for suppression of dominant plant species, benefitting rare species in coastal salt marshes, thus increasing biodiversity [23]. Recently, the North American dodder species *Cuscuta campestris* was also found on several occasions between 2018 and 2021 in sand dunes in the Bulgarian coastal area, showing an ability to adapt to salinity in its growth environment as well as drought (Figure 3). Hosts included *Centaurea arenaria* Willd., *Peucedanum obtusifolium* Sm. and *Medicago marina* L. This is posing a serious concern as this introduced, invasive species may be significantly harmful to the vulnerable ecosystems of coastal areas. *Plicosepalus acaciae* was found to infect both halophytes and glycophytes with equal success [54]. Among the root parasites, *Cynomorium coccineum* L. is an example of a typical halophytic parasitic plant of the Mediterranean [78].

Salt stress leads to a wide range of physiological and biochemical changes, which may differ significantly between salt-sensitive and salt-tolerant species. These include mineral imbalance, mostly altered Na^+^/K^+^ ratios, osmotic stress, elevated rates of reactive oxygen (ROS) production and redox changes, alongside the accumulation of compatible solutes as sugars, polyols and free amino acids such as proline [79]. Although parasitic plants have limited (root parasites) or absent (stem parasites) soil contact, they are affected by salt stress indirectly, via the metabolism of the host (Figure 2). Some parasites may prefer stressed hosts, which accumulate low-molecular-weight compounds, making them a rich source of nutrients [40]. Others may not discriminate between salt-tolerant and salt-sensitive hosts and grow on whatever is available, e.g., *Plicosepalus acaciae* (mistletoe) [54].

Salinity appears to affect parasitic plants on three main levels (Figure 2): firstly, directly in the seed germination phase; secondly, indirectly through changes in host signaling, affecting seed germination of the parasitic plant; and thirdly, indirectly through the effects of salinity on host susceptibility to parasitism. Various authors showed that salt stress significantly inhibited seed germination of parasitic plants, for example in *Orobanche cernua* [80], *Orobanche minor*, *Orobanche crenata* and *Striga hermonthica* [81]. As outlined above, in parasitic plants seed germinability may also depend strongly on the concentration of germination stimulants. Addition of the synthetic strigolactone analogue GR24 partially alleviated the negative effects of 50 mM and 75 mM NaCl on *Orobanche minor* germination [81]. Intricate interactions exist between the effects of salt concentration on seed germination of parasitic plants and arbuscular mycorrhizal fungi which suppress the production of strigolactones once the symbiosis is established. Mycorrhized *Lactuca sativa* plants were able to adapt better to salinity, and in the absence of NaCl, their root exudates were less inductive to *Orobanche ramosa* seed germination. In turn, in the presence of NaCl, exudates stimulated seed germination of *Orobanche ramosa* in a similar way as in non-mycorrhized plants [31].

It is also poorly studied if parasitic plants grow well on salt-stressed hosts. Demirbas reported increased susceptibility of *Arabidopsis* to *Orobanche ramosa* infection at 50 mM NaCl [82]. In contrast, Al-Khateeb [83] reported significantly reduced infection of *Lycopersicon esculentum* plants by *Orobanche cernua* at 50 mM and completely absent infection at 75 mM NaCl. In *Vicia faba*, several salt-tolerant cultivars were also found to be resistant to *Orobanche crenata* infection [46]. The growth of *Cuscuta campestris*, infecting salt stressed *Arabidopsis* plants was reduced by nearly 50% already at 50 mM NaCl [84]. Still, little is known about how the parasites respond to salinity. In terms of compatible solutes, some members of the Orobanchaceae family were found to synthesize polyols (mainly mannitol) [85], whereas mistletoes were able to actively absorb polyols from the host and develop a host-specific polyol profile [86]. In the case of *Cuscuta campestris*–an *Arabidopsis* parasite-host pair—, it was found that at higher salt concentrations the parasite accumulated L-proline, accompanied by decreased concentrations in the host compared to non-infected plants [84]. Despite plant response to salinity being widely studied and numerous mechanisms being well understood [87], these mechanisms have been poorly studied or not studied at all in parasitic plants.

### 3.3. Heavy Metal Stress

Heavy metals are among the most toxic compounds that affect plant metabolism and are major pollutants in arable lands, especially in areas with industrial manufacturing and mining. As reviewed elsewhere [88], plant response to heavy metals varies greatly; some plants are extremely sensitive, some show a degree of resistance and others can even accumulate or hyperaccumulate certain elements such as cadmium [89], lead [90] and nickel [91]. With regard to parasitic plants important questions are (1) whether parasitic plants acquire heavy metals from their hosts; (2) if so, whether they are able to selectively exclude at least some of them; (3) whether parasitic plants have their own protection mechanisms; and (4) whether parasitic plants comprise a potential threat to bioremediation of soils by suppressing the growth of plant hyperaccumulators.

The scarce publications available are mostly case studies rather than systematic approaches to answer these questions. An additional concern is the medicinal value of numerous parasitic plants, extracts of which are used as herbal remedies. In 2013 in Pakistan, the root parasites *Cistanche tubulosa* (Schenk) Wight and *Orobanche ramosa* were tested for their heavy metals load and, compared to several other medicinal plants [92], showed the highest concentrations of Zn, Co and Mn. According to this study, parasitic plants accumulated higher concentrations of heavy metals than all other plants studied. The host range from which the two parasites were collected was not specified, which makes it impossible to assess whether heavy metal accumulation by parasitic plants is host-specific, and it remains unclear whether direct acquisition from soil occurs.

A more detailed study of the host-root hemiparasite pair *Cistus* spp.–*Odontites lutea* Clairv. [52] contradicted earlier reports [93] that *Odontites* spp. are sensitive to heavy metals and do not occur on metalliferous soils. In polluted areas, this hemiparasite accumulated significantly lower Fe and Zn concentrations, but equal or slightly higher concentrations of Cu and Pb (Table 2). This apparent selectivity to heavy metal uptake was also reported in the stem parasite *Cuscuta campestris* on *Daucus carota*, where similar or higher concentrations of Zn and Cu and exclusion of Cd were found [53]. A striking host-specific heavy metal accumulation was reported for *Cuscuta californica* Hook. & Arn., parasitizing Ni hyperaccumulator *Streptanthus polygaloides* A. Gray and the non-accumulating host *Lessingia nemaclada* Greene [94]. The Ni concentration in the parasitic plant was considerably higher when the parasite grew on the hyperaccumulating host (compared to a non-hyperaccumulating host), but lower than that of the host itself (Table 2). Copper and Cr concentrations were equal to the respective value in the hosts. However, Co and Pb accumulated to higher values than in the host when parasitizing the non-accumulating host and lower than in the host when parasitizing the Ni hyperaccumulator. Clearly, the lack of extensive studies does not allow a conclusive overview. So far, it can be concluded that the transfer of heavy metals from the host to the parasite seems to be host- and element-specific, also depending on the parasitic plant species.

The response of parasitic plants to heavy metals is poorly understood. One of the first papers on heavy metal toxicity in parasitic plants reported that six heavy metals tested on *Cuscuta reflexa* had detrimental effects above 0.5 µg mL^−1^ [95]. Other papers showed effective heavy metal detoxification in the parasitic *Euphrasia* spp., but high sensitivity of *Odontites* spp. and *Rhinanthus* spp. [93]. More recently, Cd was shown to be toxic to in vitro cultures of *Cuscuta reflexa*, with significant inhibition of growth, shoot length and seed germination [96].

In plants, phytochelatins (PC) confer protection from heavy metal toxicity [97], and their synthesis was reported in *Cuscuta* spp. on several occasions. When *Cuscuta reflexa* was exposed to Cd, the activities of catalase, peroxidase and glutathione reductase increased up to a concentration of 300 µM and decreased at 500 µM Cd [96]. Phytochelatin synthesis occurred only in Cd-treated callus cultures and seedlings and increased dramatically at higher concentrations, up to 7-fold at 500 µM Cd, in seedlings. Furthermore, constitutive expression of PC synthase and increase in PC concentrations upon exposure to low Cd concentrations (36 µM) was reported in *Cuscuta campestris* on *Daucus carota* [53]. The authors suggested a substantial role for phytochelatins not only in heavy metal detoxification, but also in the homeostasis of essential metals in *Cuscuta* spp.—a challenging task for a parasite—, which is entirely dependent on the host for the acquisition of mineral nutrients. As there is extensive molecular trafficking between hosts and parasites [8,98] it is not unlikely that phytochelatins or other thiol compounds are transferred from the host to the parasite, but we did not find any reports on this topic.

Finally, the potential role of heavy metals to confer resistance to hyperaccumulating hosts against parasitic plants was also studied. The “elemental defense hypothesis”, reviewed by Poschenrieder [99], suggests that heavy metals protect plants against herbivores, fungal and bacterial pathogens, a potential evolutionary advantage for hyperaccumulators. Apparently, at least some parasitic plants are not affected by heavy metals in their hyperaccumulating hosts. As mentioned above, *Cuscuta californica* successfully infested the Ni hyperaccumulator *Streptanthus polygaloides* [94]. Similarly, the root parasite *Orobanche nowackiana* Markgr. was found to be a major pest on another Ni hyperaccumulator, *Alyssum murale* Waldst. & Kit. [100]. Moreover, some parasitic plants like *Orobanche lutea* may even benefit from heavy metal pollution and thrive better in such areas, simultaneously providing beneficial unload of toxic elements from the host [101], as such areas are characterized by lower competition and heavy metal accumulation provides herbivore defence [102]. More research is needed before informed conclusions can be drawn regarding the role of heavy metals in the interaction between hosts and parasitic plants, and the knowledge gained could benefit bioremediation programs.

### 3.4. Herbicide Resistance

Herbicide treatment is one of the oldest approaches to control parasitic plants in arable lands, unfortunately with questionable effectiveness. Attempts to control parasitic plants include the application of systemic herbicides at doses that are non-lethal to crops, treatment with substances that specifically target the parasitic plant species and the use of herbicide-resistant transgenic crops. The herbicide might be preferably applied immediately after germination or the initial attachment of the parasite and/or it must be transferred from the host to the parasite before being detoxified [103]. The efforts to combat parasitic plants are enormous [20], considering that the economic losses, caused by Striga spp. only account for $7 billion per year in Sub-Saharan Africa alone [104].

Commonly used herbicides can be effectively applied to control the root parasites *Striga* spp. and *Orobanche* spp. [105], but the stem parasites *Cuscuta* spp. are more challenging. Nadler-Hassar [51] showed that *Cuscuta campestris* is even more resistant to herbicides that inhibit amino acids biosynthesis than transgenic herbicide-resistant crop plants. Notably, the I_50_ value (defined as the rate in g ha^−1^, causing 50% reduction in tissue elongation) of glyphosate-treated *Cuscuta* was eight-fold higher than that of glyphosate-resistant cotton (*Gossypium hirsutum* L., cv. DP5415RR). Similar results were obtained for seven other herbicides. In another study, three *Cuscuta* species were shown to be more resistant to glyphosate and imazamox and equally resistant to glufosinate compared to either wild type or transgenic crop plants, with the exception of glufosinate-resistant oilseed rape, which showed several-fold higher I_50_ values when exposed to glufosinate than all *Cuscuta* species tested [106]. However, *Cuscuta campestris* was not substantially affected by glufosinate when parasitizing on glufosinate-resistant oilseed rape. A possible answer to this phenomenon may lie in the protein trafficking between hosts and parasites, as shown in the case of *Cuscuta pentagona*, parasitizing on transgenic soybean, where the glufosinate detoxifying phosphinothricin acetyl transferase enzyme appeared also in the parasite [107]. Thus, the parasitic plant acquired resistance from the host. It should be noted that such resistance could also be acquired through mRNA transfer into the parasite, not detected in the above study, but conceivable considering the extensive trafficking of RNAs [108] and through horizontal gene transfer, which is also common in parasitic plants [109].

## 4. Agricultural Aspects of Host-Parasite Interactions under Abiotic Stress

Parasitic plants exert major effects on the host by tapping into the host’s nutrients and photosynthates, thus restricting host growth and development. In addition, they may alter the photosynthetic performance of their hosts. Some reports showed that the host’s photosynthetic capacity is increased in order to compensate for the organic compounds sink from the host to the parasite [74]. By contrast, stomatal conductance, photosynthetic rates and carboxylation efficiency severely decreased in *Mikania micrantha* Kunth infected with *Cuscuta campestris* [110] and this effect was exacerbated by drought in the case of *Cuscuta australis* infection [111]. The negative effect of *Orobanche ramosa* infection on *Lycopersicon esculentum* was also largely attributed to the inhibition of photosynthesis rather than simple exhaustion of nutrients [112].

In a recent report we showed that *Cuscuta campestris* infection caused differential effects on different organs of the parasitized host plant [84]. The effects of infection on antioxidant enzymes were most pronounced at the infection site (direct effect), but also substantial in non-infected aerial parts and roots (indirect effects). Parasitism by *Cuscuta* further interfered with the host’s ability to accumulate osmoprotectant L-proline and to properly respond to salt stress. Similarly, the root parasite *Orobanche aegyptiaca* showed a negative effect on the salt stress response of the host, *Lycopersicon esculentum* [113].

The influence of parasitic plants on their hosts is not limited to simple exhaustion of nutrients. It was reported that parasitic plants could also manipulate host metabolism to serve their own needs. For examples, the root hemiparasite *Phtheirospermum japonicum* was found to transfer cytokinins into the host to cause root hypertrophy [114]. Moreover, *Cuscuta* spp. can actively transfer microRNAs into theirs hosts, targeting mRNAs in order to improve nutrient uptake [115,116,117]. So far, it remains unknown whether the pattern of host manipulation is affected by abiotic stress factors. However, evidence was provided that *Cuscuta* spp. may be responsible for transfer of stress signals between simultaneously infected hosts, thus affecting positively response to salt stress [118], which was also previously shown for herbivore-induced signals [119].

Crop infestation by parasitic plants is highly detrimental to agricultural plant communities, leading to severe yield reduction or even complete crop loss. It may exacerbate the effects of abiotic stress (and vice versa). For example, *Vicia faba* (fava bean), a major grain legume that is often cultivated on saline soils and irrigated with diluted sea water (e.g., in Egypt), is susceptible to *Orobanche crenata* infestation, which may lead to complete loss of yield [120]. Broomrapes also cause crop losses in areas characterized by arid and saline soils in Sub-Saharan Africa and Israel [121]. Fernández-Aparicio reported a case of acquired susceptibility of *Lupinus albus* L. (white lupin) to infection with *Orobanche crenata* when the crop plant was cultivated on alkaline soils [122]. In several cases, however, abiotic stress tolerance of selected cultivars coincided with insusceptibility to parasitic plant. Notable examples are salt- and broomrape-tolerant *Vicia faba* cultivars [46] and drought-tolerant and witchweed-resistant *Zea mays* L. cultivars [123]. Again, more research is needed to better understand the effects of abiotic stress factors on the interactions between host and parasitic plants, especially with a view to climate change, the ever-increasing threats of which to agricultural production can be exacerbated by parasitic plants.

## 5. Challenges and Outlook

The interpretation of data on parasitic plants generally, and with regard to their response to abiotic stress factors specifically, is challenging due to the wide spectrum of hosts studied. The situation is further complicated by the distinct features of root and stem parasites [1] and the apparent influence of symbiotic microorganisms in the case of root parasites [31]. In order to acquire large sets of comparable data on the response of parasitic plants to abiotic stress factors, a well-established model system needs to be developed. Evidence is emerging that *Arabidopsis thaliana* may be a suitable, albeit not common, host plant to study plant-plant parasitism. *Arabidopsis* is susceptible to many *Orobanche* spp. [82,124,125,126,127,128] and *Cuscuta* spp. [129,130] and could be used to study interactions between parasitic plants and their hosts regarding germination stimulants, haustoria-inducing factors and host response to parasitism [131]. The ease of acquiring *Arabidopsis* mutants and the possibility to identify parasite-resistant genotypes is a further bonus of this system. With regard to salt tolerance, *Arabidopsis thaliana* and *Eutrema halophilum* represent a promising glycophyte/halophyte pair of closely related host species for future research [41], but it is still to be confirmed if *Eutrema halophilum* is susceptible to parasitic plants.

Most research into parasitic plants has been directed towards economically significant parasites, but knowledge of stress-tolerant parasitic plants is lacking. The latter are interesting evolutionary cases [1] but may represent a major threat to agricultural production in times of climate change and also to natural ecosystems [22]. The study of stress-tolerant parasitic plants may be compromised by their low biomass production, but this could be overcome by the development of in vitro cultures of parasitic plants [132,133]. Another future focus should be on stress-induced changes in hormones involved in response to environmental stress factors, such as abscisic acid, jasmonic acid, salicylic acid and ethylene [134] in parasitic plants. 

In conclusion, parasitic plants represent a fascinating line of evolution, but can compromise agricultural production. Despite the studies available on the molecular mechanisms of plant parasitism and the ecological and agricultural impacts of this highly specialized group of angiosperms, knowledge gaps exist regarding their response to abiotic stress factors. Parasitic plants are not uncommon in challenging environments, either saline, arid, polluted or cold. Suboptimal conditions may alter their host preference, and stress effect and response may be host-dependent or -independent. To better understand their potential impacts on contemporary agriculture in times of climate change, a more systematic approach is needed, requiring the development of suitable models of stress-tolerant and stress-sensitive pairs of hosts and parasitic plants. More data on the plastid, mitochondrial and nuclear genomes sequences would also be helpful. We hope that this review helps to raise awareness for and stimulate more research on parasitic plants and their multiple facets from representing agricultural pests to being important members of plant communities and intriguing models to study plant-plant interactions.

## Figures and Tables

**Table 1:** Examples of parasitic plants and their respective hosts growing in environments characterized by abiotic stress pressure such as low temperatures, water deficiency and high soil salinity.

Parasitic Plant	Classification	Distribution	Host	References
**Arctic environment**				
*Bartsia* spp. *Euphrasia* spp. *Pedicularis* spp.	Root hemiparasite	North of 70° N latitude	Various shrubs	[55]
**Environment characterized by frequent droughts**				
*Cistanche deserticola* Ma. *Cistanche tubulosa* (Schrenk) Hook.f.	Root holoparasite	Central Asia, Middle East	*Haloxylon* spp.,	[56]
*Cistanche phelypaea* (L.) Cout.	Root holoparasite	Mediterranean, Arabian Peninsula	*Atriplex* spp., *Nitraria* spp.	[57]
*Hydnora africana* Thunb.	Root holoparasite	South Africa	*Euphorbia* spp.	[58]
*Tristerix aphyllus* Miers	Stem holoparasite	South America	Various cacti	[59]
**Environment characterized by salinity**				
*Cuscuta salina* Engelm., *Cuscuta pacifica* Costea and M.A.R. Wright	Stem holoparasite	North America, mainly the Pacific coast	*Frankenia* spp.*, Suaeda* spp.*, Salicornia* spp.*, Jaumea* spp.	[60]
*Cuscuta tasmanica* Engelm.	Stem holoparasite	Southern Australia and Tasmania	*Wilsonia* spp., *Sarcocornia* spp.	[61]
*Cynomorium coccineum* L., including var. *songaricum*	Root holoparasite	Mediterranean, Central Asia	*Atriplex* spp., *Nitraria* spp.	[62,63]
*Plicosepalus acacia* (Zucc.) Wiens & Polhill	Stem hemiparasite	North-Eastern Africa, Arabian Peninsula, Middle East	*Atriplex* spp., *Tamarix* spp., *Nitraria* spp.	[54]

**Table 2:** Transfer of various elements from hosts to parasitic plants. No simple relationship exists for the proportion of chemical elements found in hosts and parasites: according to the scarce data available in the literature, a certain element in the same parasite can apparently range from less than 10% of the host concentration up to almost 1000-fold, corresponding to a ratio of concentration in the parasite divided by the concentration in the host of 0.1 to almost 10.

Parasite-Host Pair	Ratios of Concentrations (Parasite/Host)	Note	Reference
*Cuscuta californica*-multiple	Ni: 0.3–0.6 Cu: 0.9–1.3 Zn: 0.9–1	Depending on host species	[94]
*Cuscuta campestris*-*Daucus carota*	Cd: 0.1–0.4 Zn: 0.4–5.3 Cu: 0.5–1	Depending on duration of treatment and host organ	[53]
*Odontites lutea*-*Cistus* sp.	Pb: 0.6–1.3 Cu: 1.2–1.8 Zn: 0.6–0.9	Depending on soil pollution	[52]
*Plicosepalus acaciae*-multiple	Na^+^: 0.3–9.4 Cl^−^: 0.3–5.1	Depending on host species and host organ	[54]

## Data Availability

Not applicable.
